# Clinical utility of markerless motion capture for kinematic evaluation of sit-to-stand during 30 s-CST at one year post total knee arthroplasty: a retrospective study

**DOI:** 10.1186/s12891-023-06364-3

**Published:** 2023-04-01

**Authors:** Katsuya Onitsuka, Keisuke Kubota, Moeka Yokoyama, Taku Miyazawa, Toyohiro Hamaguchi, Hiroto Taniguchi, Naohiro Usuki, Satoshi Miyamoto, Ken Okazaki, Kenji Murata, Naohiko Kanemura

**Affiliations:** 1 Department of Rehabilitation, Yachiyo Medical Center, Tokyo Women’s Medical University, Chiba, Japan; 2 Graduate Course of Health and Social Services, Graduate School of Saitama Prefectural University, Saitama, Japan; 3 Department of Physical Therapy, School of Health and Social Services, Saitama Prefectural University, 820 Sannomiya, 343-8540 Saitama, Japan; 4 Sportology Center, Juntendo University Graduate School of Medicine, Tokyo, Japan; 5 Department of Orthopaedic Surgery, Ushiku Aiwa General Hospital, Ibaraki, Japan; 6 Department of Orthopaedic Surgery, Yachiyo Medical Center, Tokyo Women’s Medical University, Chiba, Japan; 7 Department of Orthopaedic Surgery, Tokyo Women’s Medical University, Tokyo, Japan

**Keywords:** Sit-to-stand, Kinematics, Chair stand test, Markerless motion capture, Total knee arthroplasty, Forgotten joint

## Abstract

**Background:**

Although the importance of kinematic evaluation of the sit-to-stand (STS) test of total knee arthroplasty (TKA) patients is clear, there have been no reports analyzing STS during the 30-s chair sit-up test (30 s-CST) with a focus on kinematic characteristics. This study aimed to demonstrate the clinical utility of kinematic analysis of STS during the 30 s-CST by classifying STS into subgroups based on kinematic parameters, and to determine whether differences in movement strategies are expressed as differences in clinical outcomes.

**Methods:**

The subjects were all patients who underwent unilateral TKA due to osteoarthritis of the knee and were followed up for one year postoperatively. Forty-eight kinematic parameters were calculated using markerless motion capture by cutting STS in the 30 s-CST. The principal components of the kinematic parameters were extracted and grouped by kinematic characteristics based on the principal component scores. Clinical significance was examined by testing whether differences in patient-reported outcome measures (PROMs) were observed.

**Results:**

Five principal components were extracted from the 48 kinematic parameters of STS and classified into three subgroups (SGs) according to their kinematic characteristics. It was suggested that SG2, using a kinematic strategy similar to the momentum transfer strategy shown in previous studies, performed better in PROMs and, in particular, may be associated with achieving a “forgotten joint”, which is considered the ultimate goal after TKA.

**Conclusions:**

Clinical outcomes differed according to kinematic strategies used STS, suggesting that kinematic analysis of STS in 30 s-CST may be useful in clinical practice.

**Trial registration:**

This study was approved by the Medical Ethical Committee of the Tokyo Women’s Medical University (approval number: 5628 on May 21, 2021).

**Supplementary Information:**

The online version contains supplementary material available at 10.1186/s12891-023-06364-3.

## Background

Osteoarthritis of the knee is a typical joint disease in older adults and, in severe cases, total knee arthroplasty (TKA) is indicated, with the aim of allowing the patient to return to society by improving physical function through physical therapy thereafter. To determine a treatment plan, it is important to evaluate physical function using a kinematic analysis of motion and to clarify the picture of disability [1].

The Osteoarthritis Research Society International (OARSI) recommends patient-reported outcome measures (PROMs) and performance-based tests to assess the physical function of patients undergoing TKA [2]. The 2011 ver Knee Society Score (KSS) [3] and the Knee Injury and Osteoarthritis Outcome Score (KOOS) [4] are commonly used in PROMs to evaluate functional impairment. In addition, the Forgotten Joint Score-12 (FJS-12) [5] was developed based on a final goal after artificial joint surgery of the patient’s being able to live daily life without being aware of the operated joint (it has become a "forgotten joint"). The number of reports using it in the field of artificial joints has been increasing in recent years.

Performance-based tests reflect a patient’s ability to perform real activities and include three assessments in the minimum core set: sit-to-stand (STS); gait; and stair climbing. Among these, the 30-s chair stand test (30 s-CST) [3] is easier to use in clinical practice than other assessments because it can be performed in a small space such as an examination room. Additionally, it is highly reliable and reproducible [6], has excellent correlation with walking ability [7], and is an indicator of the risk of falls [8]. Therefore, STS testing is indicated for patients undergoing TKA [9]. In addition, previous studies on kinematic changes in the STS in TKA patients have reported that the kinematic strategies used by a patient that were observed preoperatively continue to be observed one year after TKA [10], and that these changes not only decrease motor efficiency but may also cause new symptoms in other parts of the body such as the hip [10]–[12]. The importance of kinematic analysis of STS is clear.

On the other hand, because the 30 s-CST only captures movement speed, it does not reveal the underlying mechanisms that cause functional impairment and has low correlation with patient-reported outcome measures (PROMs) [13]. To identify the underlying mechanisms causing functional impairment and understand the disability picture of the patient as indicated by PROMs, the STS during the 30 s-CST must be analyzed in detail from a kinematic point of view [14]. However, the mechanisms related to kinematic changes in STS are complex and have been shown to be influenced not only by disease (e.g., knee osteoarthritis [15] and TKA [16]) but also, significantly, by neuromusculoskeletal physiological capacity. Additionally, when the task demands exceed one’s own capacity, STS kinematics are recognized to change [17, 18]. Therefore, the 30 s-CST, which is to be performed under defined environmental conditions and at maximum speed, is not only an evaluation of STS ability in daily life but also reflects the physical ability of the entire body to change and adapt in response to task demands. However, because these kinematic analyses require a three-dimensional motion analysis device, they can only be performed in a limited environment such as a laboratory, and the burden on the patient is high. Therefore, they have not been widely used in clinical practice, where therapists often perform kinematic analysis of STS based on observation and experience. The lack of a scientific basis for the evaluation of results has been an issue for many years.

We thought that the clinical application of a markerless motion capture system could solve this problem. The markerless motion capture system can obtain kinematic data by automatically extracting the position of each joint from video captured by a monocular camera. Relative to 3D motion analyzers, which are the gold standard for motion analysis, disparate definitions for calculation of segment and joint angles and errors in kinematic data calculations from tracking errors are issues to be addressed. However, its very high reproducibility and low burden on the patient have led to clinical application of the markerless motion capture system. It has been used for motion analysis in the orthopedic field and reported to be effective in classifying healthy subjects and patients with knee disease [19] and in predicting the moment of knee adduction during gait [20]. However, there have been no reports of clinical application to post-TKA patients, and the relationship between kinematic analysis results and clinical outcomes has not been clarified.

Based on the above, we believed that analysis of the STS in the 30 s-CST in TKA patients using markerless motion capture with a focus on kinematic characteristics could provide a new indicator of postoperative functional recovery and contribute as a medical aid when determining physical therapy treatment plans. We aimed to analyze the kinematics of STS during the 30 s-CST at one year after TKA to develop the following three items: 1) a procedure for principal component analysis enabling extraction of the principal components through contraction of the dimensions of STS kinematic parameters calculated by markerless motion capture; 2) a classification of the STS during the 30 s-CST as performed one year after TKA based on a cluster analysis of the extracted principal components of the kinematic parameters; 3) a method to demonstrate the clinical utility of kinematic analysis of the STS in the 30 s-CST through identification of how clinical outcomes are related to the classification results.

## Methods

### Subjects

All patients who underwent unilateral TKA at Tokyo Women’s Medical University Yachiyo Medical Center for knee osteoarthritis between April 2017 and March 2021 and were followed up for one year postoperatively were included. In this study, patients who received bilateral TKA and those who had difficulty performing 30 s-CST (including those who had difficulty performing the movement with the upper extremities crossed in front of the chest) were excluded because the aim was to determine the association between the results of STS classified by kinematic characteristics and clinical outcomes. Although the duration and frequency of the interventions varied from patient to patient, all patients received standard physical therapy interventions aimed at improving lower limb function and ADL. The study was conducted with the approval of the Ethics Committee of the Tokyo Women’s Medical University (approval number: 5628 on May 21, 2021). All patients provided informed consent via opt-out.

### Characteristics

Age, sex, body mass index (BMI), femorotibial angle (FTA), knee joint range of motion (ROM), and knee joint extensor strength were obtained from medical records. Knee joint ROM was measured using a standard goniometer (Kaminaka type angle meter; Sakai Medical Corporation, Tokyo, Japan) in accordance with the standards of the Japanese Orthopaedic Association and the Japanese Society of Rehabilitation Medicine. Knee joint extensor muscle strength was measured using a manual muscle tester (μTasMT-1; Anima Corporation, Tokyo, Japan) to measure the isometric knee joint maximum extension muscle strength of the affected side. The patient sat on the edge of the bed, with both upper limbs crossed in front, and the trunk kept in a vertical position. The sensor pad was placed on the front of the distal shank and fixed to the bed leg by adjusting the length of the belt, such that the shank was in a drooping position. Isometric knee joint extension exercises were performed at maximum effort for approximately 5 s, twice with a measurement interval, and the average value was adopted. The value, divided by body weight, was used for analysis.

### Performance-based test

The 30 s-CST, 8-step stair climbing test (8-step SCT), and 40 m fast-paced walk test (40 m FPWT), which OARSI recommends being performed as the minimum core set, were conducted in accordance with standardized methods [2]. The 30 s-CST used a chair with a backrest and no armrests, with a seat height of 17 inches (44 cm). The test limb was positioned with the feet shoulder-width apart, knees slightly more than 90° flexed, and arms crossed in front of the chest. The subjects were instructed to stand up completely from a sitting position, the hip and knee joints fully extended, and to be fully grounded to the chair when seated; they repeatedly stood up and down from the chair as fast as possible for 30 s, and the number of times they were able to stand up and down completely was recorded. If the subject was unable to stand once under the above conditions, including by placing hands on the feet and using aids, the score was set to 0.

The 8-step SCT was performed on a staircase with a 16.5 cm kick-up and handrail. The number of steps was not specified in the test protocol; the test was performed in eight steps owing to the structure of the hospital. The use of handrails and walking aids was permitted, if necessary; however, the patient was instructed to ascend and descend as quickly as possible. The measurement started at the start signal, and the time taken for both feet to return to the starting point was measured.

The 40 m FPWT was conducted on a 10-m walking path that provided a safe space for changing directions. The participants were instructed to walk the path four times in succession at the fastest speed possible, although walking aids used in daily life were permitted. The measurements were started at the start signal, the time taken to cross the start point was measured, and the distance divided by the time was recorded.

### Kinematic parameters of STS

A standard digital video camera (IXY210, Canon Inc., Tokyo, Japan; IXY180, Canon Inc., Tokyo, Japan; EX-ZS210, CASIO, CASIO COMPUTER CO., LTD, Tokyo, Japan) was used to capture STS motion (30 fps).

**Fig. 1:**
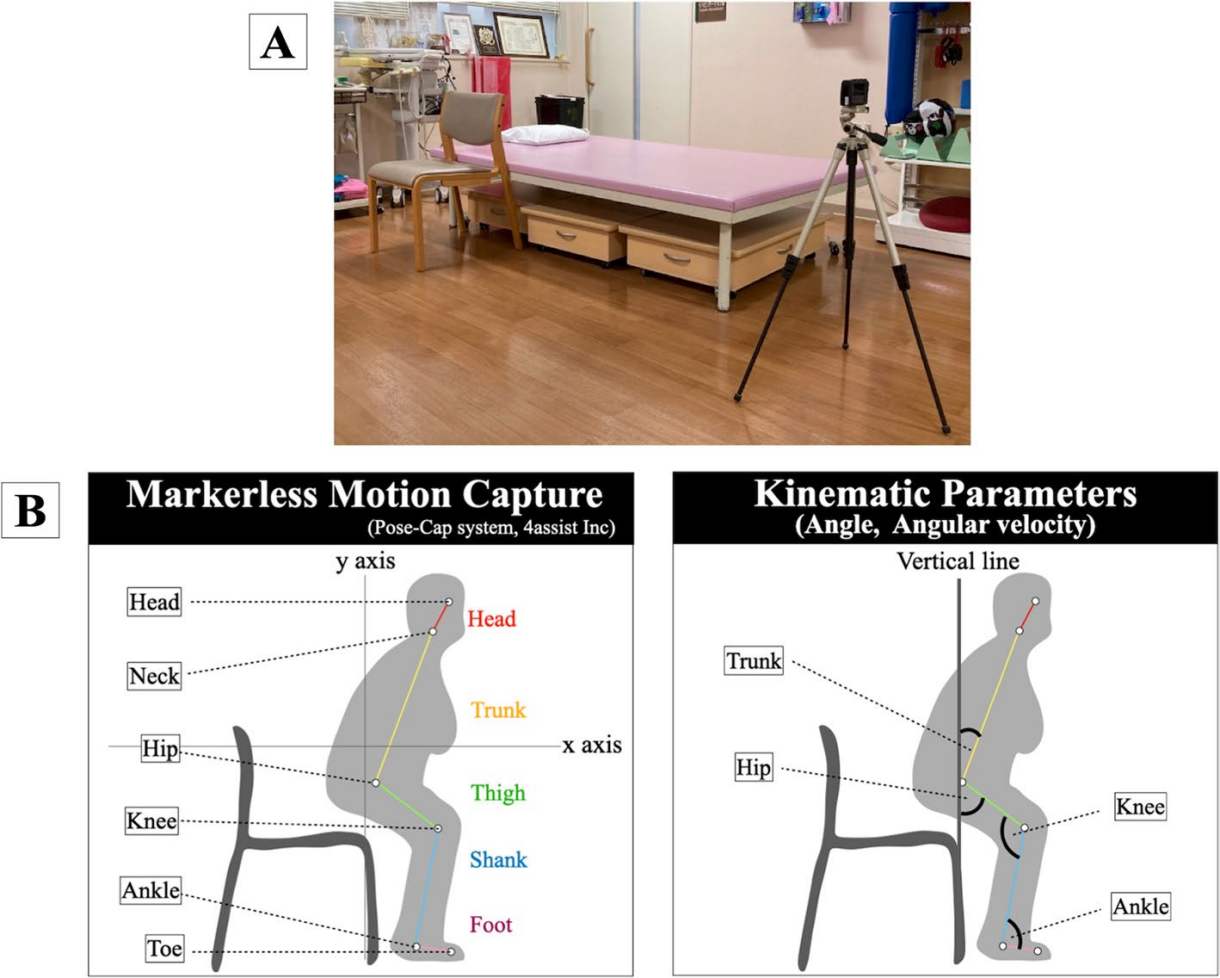
Kinematic parameter analysis method. **A** The camera was set up so that the whole-body image was centered from the operative side, and a level on a tripod was used to ensure that it was parallel to the floor and that no person overlapped the subject's background. **B** Definition of the parts and segments automatically tracked by markerless motion capture and the calculation of joint angle and angular velocity

Regarding the recording settings, previous studies using markerless motion capture have made detailed stipulations to improve measurement accuracy, including regarding the subject's clothing and the camera height and distance from the subject [21, 22], but there are many clinical situations where it is difficult to make detailed measurement stipulations. This study applied only the following three rules: (1) the camera should be positioned so that the whole body image showing the operative side appears in the center, (2) a leveler should be used on the tripod, and (3) no person should overlap the subject's background (Fig. 1A).

The recorded video data were analyzed using Pose-Cap (4assist Inc., Tokyo, Japan), a markerless motion capture system. Pose-Cap is a body authentication program similar to Open Pose [23], which is widely used for markerless motion capture, and Pose-Cap is useful in that it is systematized to be clickable without requiring engineering knowledge, making it easy for clinicians to use. Although Pose-Cap has a low agreement rate with VICON due to different definitions of segment and joint angle calculations, it has very high reproducibility and is well adapted to capture gross kinematic features in a clinical setting (Supplemental Data 1). The markerless motion capture system automatically extracts the position of each joint, from the head to the toes, from a video captured by a monocular camera to obtain two-dimensional joint coordinates (Fig. 1B).

Segments were defined as follows: trunk, neck to the center of both hip joints, thigh, hip to knee joint, shank, knee to ankle joint, foot, ankle to toe. Joint angles were calculated with reference to the above segments and defined as follows: trunk, angle formed by the trunk axis and perpendicular to the floor; hip, angle formed by the femoral axis and perpendicular to the floor; knee, angle formed by the femoral axis and shank axis; ankle, angle formed by the shank axis and foot.

In this study, 48 parameters of motion in the sagittal plane were calculated. Two kinematic parameters (angle and angular velocity) were calculated for four joints (trunk, hip, knee, ankle) in two directions (flexion, extension). Then, the maximum value (described in the text as max angle/max angular velocity), maximum value arrival time (described in the text as max angle time/max angular velocity time), and maximum difference between the maximum values (described in the text as Max–Min max angle/Max–Min max angular velocity) were calculated respectively.

The maximum value was selected because the 30 s-CST is a test that evaluates the rising motion at maximum speed and joint motion such that a person’s body capabilities are used to the maximum extent possible. The maximum value arrival time was normalized from sitting to standing as 100%, and the time at which the maximum value of each kinematic parameter was recorded was calculated. This item was chosen to evaluate the sequential nature of each joint movement by indicating at what point during the rise movement the joint movement reached its maximum value. The maximum difference between the maximum values was the difference in kinematic parameters within each subject, a value indicating the variability of STS during 30 s-CST. This item was chosen because the 30 s-CST is a test in which the rise at maximum speed is repeated for 30 s, so this kinematic variable is used in the analysis from the beginning to the end of the test. This was selected to evaluate the presence or absence of variation in the kinematic parameters used in the analysis from the beginning to the end of the test.

The definition of STS onset and termination was based on previous studies using angular velocity of the trunk as an indicator [24], but there are no reports of definitions for repetitive STS at maximal velocity, such as in the 30 s-CST, and it is reported that determining STS onset from a limited set of variable identifiers may overlook the contribution of initial movements from other segments [25, 26]. In this study, preliminary experiments showed that the start of movement was best captured when the composite vector of the X and Y axes of the head exceeded 5 SD in 1 s at rest, and the end of movement when the Y coordinate of the head reached its maximum value and this could be visually confirmed using the 2-dimensional coordinate data of the head. Therefore, these were the definitions used in this study.

In addition, recordings of all STSs performed during the 30 s-CST procedure were clipped, and procedures with no tracking errors according to Pose-Cap were extracted. All kinematic parameter calculations were corrected using the subject's actual measured height. Values exceeding 1.5 times the limits of the interquartile range were excluded from the analysis as outliers, after which the mean was calculated and used in subsequent analyses.

### PROMs

PROMs included the KSS [3] and KOOS [4] as disease-specific assessment measures and the FJS-12 as an assessment of joint awareness [5]. The KSS was rated on a total of 180 points using only the patient entry form and consisting of four subscales: (1) knee symptoms (seven items, 100 points); (2) satisfaction (five items, 40 points); (3) expectations (three items, 15 points); and (4) activity (19 items, 100 points). Higher scores indicated better performance.

The KOOS consists of five sub-items: symptoms; pain; daily living; sports and recreational activities; and quality of life. Scoring is based on a five-choice format, with a percentage calculated for each sub-item and higher scores indicating better performance.

The FJS-12 is a self-administered questionnaire that assesses the degree of joint awareness in 12 daily activities. The FJS-12 items are 1) Awareness in bed at night, 2) Awareness sitting on a chair for more than 1 h, 3) Awareness when you are walking for more than 15 min, 4) Awareness taking a bath/shower, 5) Awareness traveling in a car, 6) Awareness climbing stairs, 7) Awareness walking on uneven ground, 8) Awareness when standing up from a low-sitting position, 9) Awareness standing for long, 10) Awareness doing housework or gardening, 11) Awareness taking a walk/hiking, and 12) Awareness doing your favorite sport. It is rated on a five-point Likert scale (0–4), with a maximum score of 100 and a minimum score of 0. A higher total score indicates a state of being able to live without joint awareness.

### Statistical analysis

Shapiro–Wilk tests were performed to test for normality. Principal component analysis was used to reduce the dimensionality of the kinematic parameters, and the Simplimax rotation method was used to estimate the principal components. To determine the number of principal components, a parallel analysis was used to adopt principal components with higher eigenvalues than the randomly generated data based on the same number of variables and the same sample size. Hierarchical cluster analysis was performed based on principal component scores, and a subgroup (SG) was formed based on the kinematic characteristics of the STS. Differences in subject characteristics and clinical outcomes between SGs were examined using one-way analysis of variance, with the Steel–Dwass test as a multiple comparison test. All analyses were performed using R.ver. 12.1.0 with a significance level of *p* < 0.05.

## Results

**Fig. 2:**
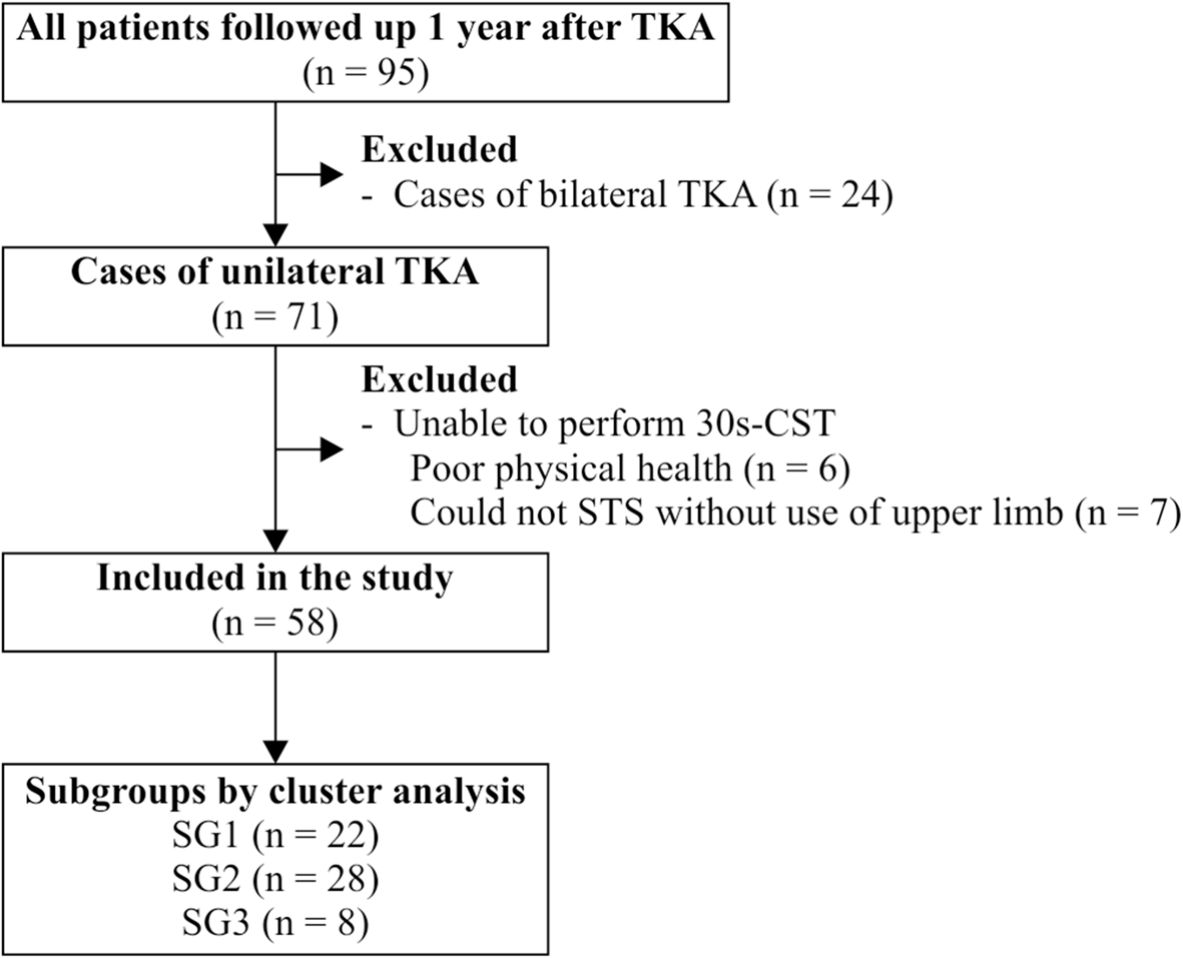
Flowchart of the included patients according to the format of the CONSORT Statement. TKA, Total knee arthroplasty; 30 s-CST, 30-s chair stand test; STS, Sit-to-stand; SG, subgroup

A total of 95 patients were included in the analysis, and 58 patients in the first year after TKA surgery fulfilled the inclusion criteria (Fig. 2). Patient characteristics are shown in Table 1.

**Table 1:** Patient characteristics

					Statistical analysis (*p*)		
	ALL (*n* = 58)	SG1 (*n* = 22)	SG2 (*n* = 28)	SG3 (*n* = 8)	SG1 × SG2	SG1 × SG3	SG1 × SG2
Age (y)	74.5 [70–79]	77.5 [71.5–79]	71.5 [67.75–79]	73 [71.5–76.25]	NS	NS	NS
Sex	F: 42, M: 16	F: 16, M: 6	F: 19, M: 9	F: 7, M: 1	NS	NS	NS
BMI (kg/m^2^)	26.05 [22.94–30.13]	26.05 [25.167–29.02]	23.89 [21.08–27.34]	30.65 [29.247–35.22]	NS	< 0.05	< 0.05
FTA (°)	174 [171–176]	173 [171–177]	174 [172–175.25]	171.5 [169.5–173.5]	NS	NS	NS
ROM flex (°)	120 [111.25–130]	125 [116.25–133.75]	120 [110–130]	120 [107.5–125]	NS	NS	NS
ROM ext (°)	0 [0]	0 [0]	0 [0]	0 [-2.5–0]	NS	NS	NS
Knee ext muscle (kg/kgf)	0.38 [0.25–0.43]	0.33 [0.24–0.40]	0.40 [0.29–0.46]	0.29 [0.21–0.38]	NS	NS	NS

Data are presented as median [IQR]. *SG* Subgroup, *BMI* Body mass index, *FTA* Femorotibial angle, *ROM* Range of motion

### Principal component analysis

**Fig. 3:**
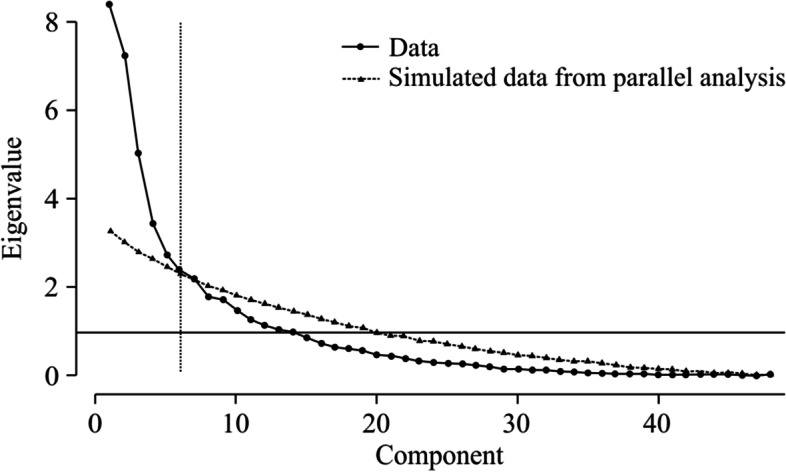
Scree plot. Based on the actual data, five principal components with larger eigenvalues than those simulated in the parallel analysis were extracted

Parallel analysis resulted in the extraction of five principal components with a cumulative contribution of 55.7%. In each principal component, characteristics were captured from the top three principal component loadings, and the principal component scores, which are parameters that indicate the characteristics of each subject, were calculated. The scree plots are shown in Fig. 3, and the principal component loadings are shown in Table 2.

**Table 2:** Results of principal component analysis

	**Principal component loading**				
	**PC1**	**PC2**	**PC3**	**PC4**	**PC5**
**Hip extension max angular velocity**	**0.86**				
**MAX–MIN hip extension max angular velocity**	**0.84**				
**Knee extension max angular velocity**	**0.80**				
MAX–MIN knee extension max angular velocity	0.77				
Knee flexion max angular velocity	0.68				
MAX–MIN knee flexion max angular velocity	0.68				
Hip flexion max angular velocity	0.66				
MAX–MIN hip flexion max angular velocity	0.61				
**Ankle dorsal flexion max angular velocity**	0.57			**0.57**	
MAX–MIN trunk extension max angular velocity	0.55				
MAX–MIN knee flexion max angle					
**MAX–MIN ankle dorsal flexion max angular velocity**				**0.55**	
**Ankle plantar flexion max angular velocity**				**0.67**	
**Trunk extension max angle time**		**-0.73**			
MAX–MIN ankle plantar flexion max angular velocity					
Trunk flexion max angular velocity time		0.67			
Ankle plantar flexion max angle					
Hip flexion max angle time		0.62			
MAX–MIN hip flexion max angle					
**Trunk flexion max angle time**		**0.87**			
**Trunk flexion max angular velocity**		**0.82**			
Trunk extension max angular velocity time		0.69			
Hip extension max angular velocity time		0.63	0.54		
**Knee extension max angular velocity time**		0.63	**0.63**		
Ankle dorsal flexion max angle time		0.63			
Hip flexion max angle		0.60			
Knee flexion max angle time		0.60			
**Trunk extension max angular velocity**					**0.59**
Trunk flexion max angle					0.51
Ankle plantar flexion max angle time					
Hip extension max angle time			0.54		
**MAX–MIN hip extension max angle**			**0.74**		
**MAX–MIN knee extension max angle**			**0.73**		
Hip extension max angle			-0.60		
MAX–MIN trunk flexion max angle			0.58		
MAX–MIN trunk flexion max angular velocity			0.54		
Knee extension max angle time					
Ankle plantar flexion max angular velocity time					
MAX–MIN ankle plantar flexion max angle					
MAX–MIN ankle dorsal flexion max angle					
**Hip flexion max angular velocity time**					**0.68**
**Knee flexion max angular velocity time**					**0.67**
Ankle dorsal flexion max angular velocity time					
Trunk extension max angle					
Knee flexion max angle					
Knee extension max angle					
Ankle dorsal flexion max angle					
MAX–MIN trunk extension max angle					

Principal component loadings greater than 0.5 were extracted. The top three principal component loadings in each principal component are in bold

The first principal component (PC1) was extracted for the hip extension maximum angular velocity, MAX–MIN hip extension maximum angular velocity, and knee extension maximum angular velocity. Higher PC1 scores can be interpreted as faster maximum angular velocity of the hip and knee joints and greater variation in the maximum angular velocity of hip joint extension.

The second principal component (PC2) involved items related to the trunk; the maximum flexion angle time, maximum flexion angular velocity time, and maximum extension angle time were extracted. A higher PC2 score can be interpreted as a slower time to reach maximum trunk flexion, a faster time to reach maximum trunk extension angle, and a faster angular velocity of maximum trunk flexion.

The third principal component (PC3) was extracted for the maximum hip extension angle, maximum knee extension angle, and maximum angular velocity time. A higher PC3 score can be interpreted as greater variation in the maximum hip and knee joint extension angles and a slower time to reach maximum knee joint extension angular velocity.

The fourth principal component (PC4) was related to ankle joint motion and was extracted as the plantar flexion maximum angular velocity, dorsiflexion maximum angular velocity, and MAX–MIN dorsiflexion angular velocity. Higher fourth principal component scores can be interpreted as higher ankle joint maximum plantar flexion and dorsiflexion angular velocities, and greater variation in ankle joint maximum dorsiflexion angular velocity.

The fifth principal component (PC5) was extracted for hip flex-maximum angular velocity time, knee flex-maximum angular velocity time, and trunk extension maximum angular velocity. A higher PC5 score can be interpreted as a faster time to reach the maximum angular velocity of hip and knee joint flexion and a faster angular velocity of trunk extension.

### Cluster analysis

**Fig. 4:**
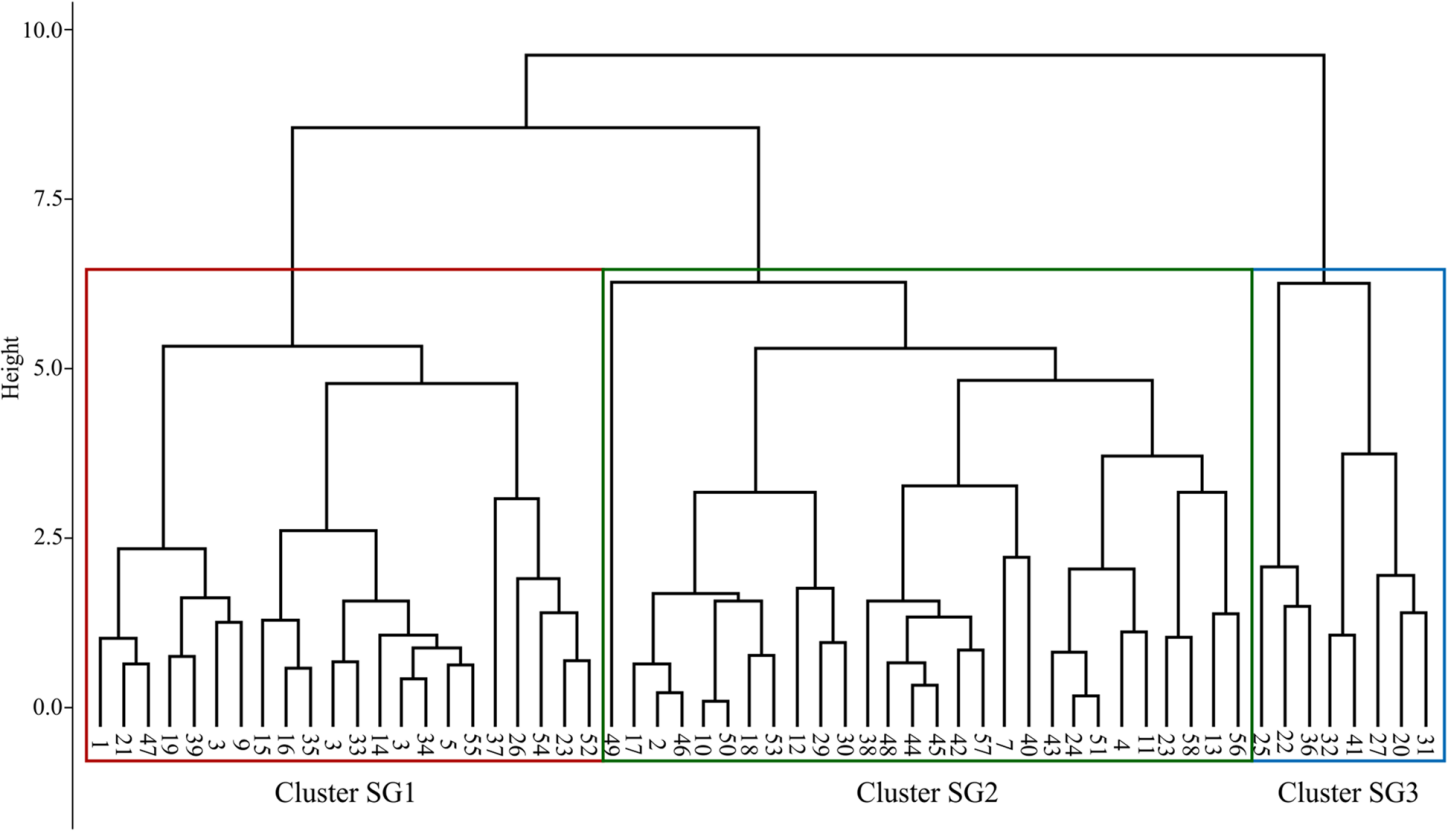
Dendrogram. Results of cluster analysis using five principal component scores. Subjects were classified into three subgroups (SG)

**Fig. 5:**
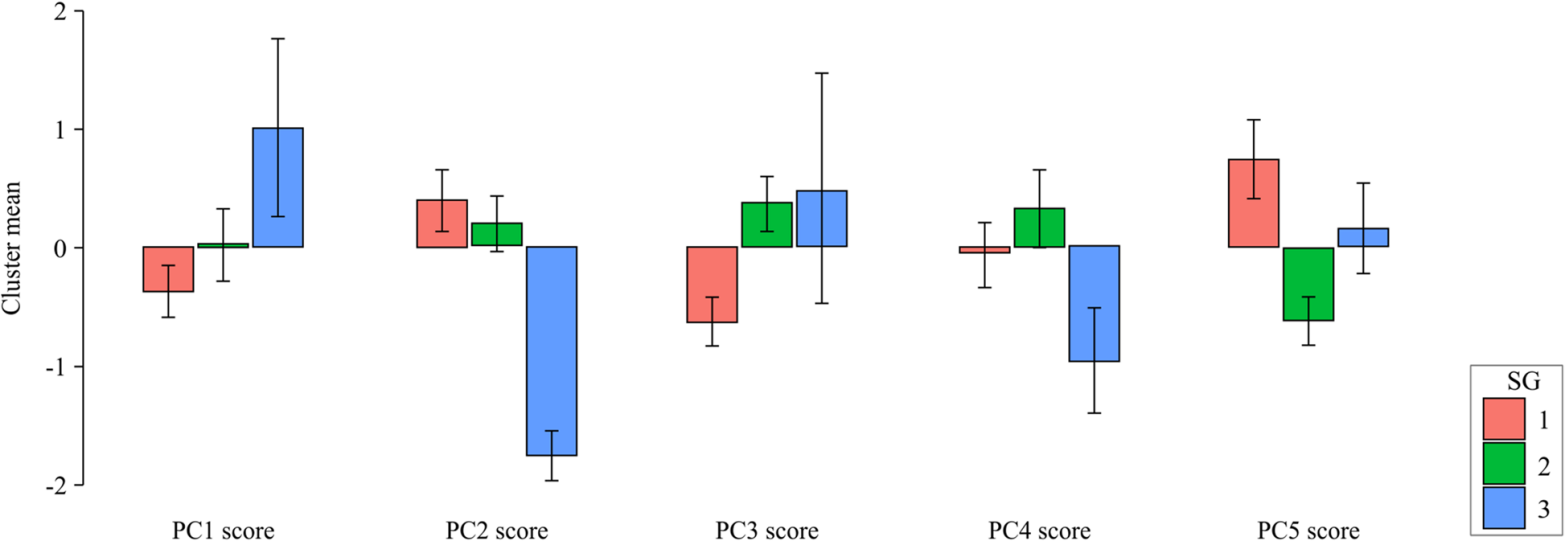
Cluster mean plots. Compared to the other clusters, SG1 had a lower PC1 score but higher PC2 and PC5 scores. SG2 had many items that fell in the middle of the SGs. SG3 had a high PC1 score and low PC2 score, symmetrical with and opposite to SG1. SG1–SG3, first to third subgroup; PC1–PC5, first to fifth principal component

A cluster analysis was performed based on the five PC scores extracted from each participant’s data. With reference to the dendrogram, the subjects were classified into three SGs (Fig. 4). SG1 had a lower PC1 score than the other clusters, whereas PC2 and PC5 scores were higher. SG2 had many items (PC1, PC2, and PC3 scores) that fell in the middle of the SGs. SG3 had a high PC1 score and a low PC2 score, opposite to SG1. SG3 was characterized by the highest PC3 score (Fig. 5).

### Difference in clinical outcome

**Fig. 6:**
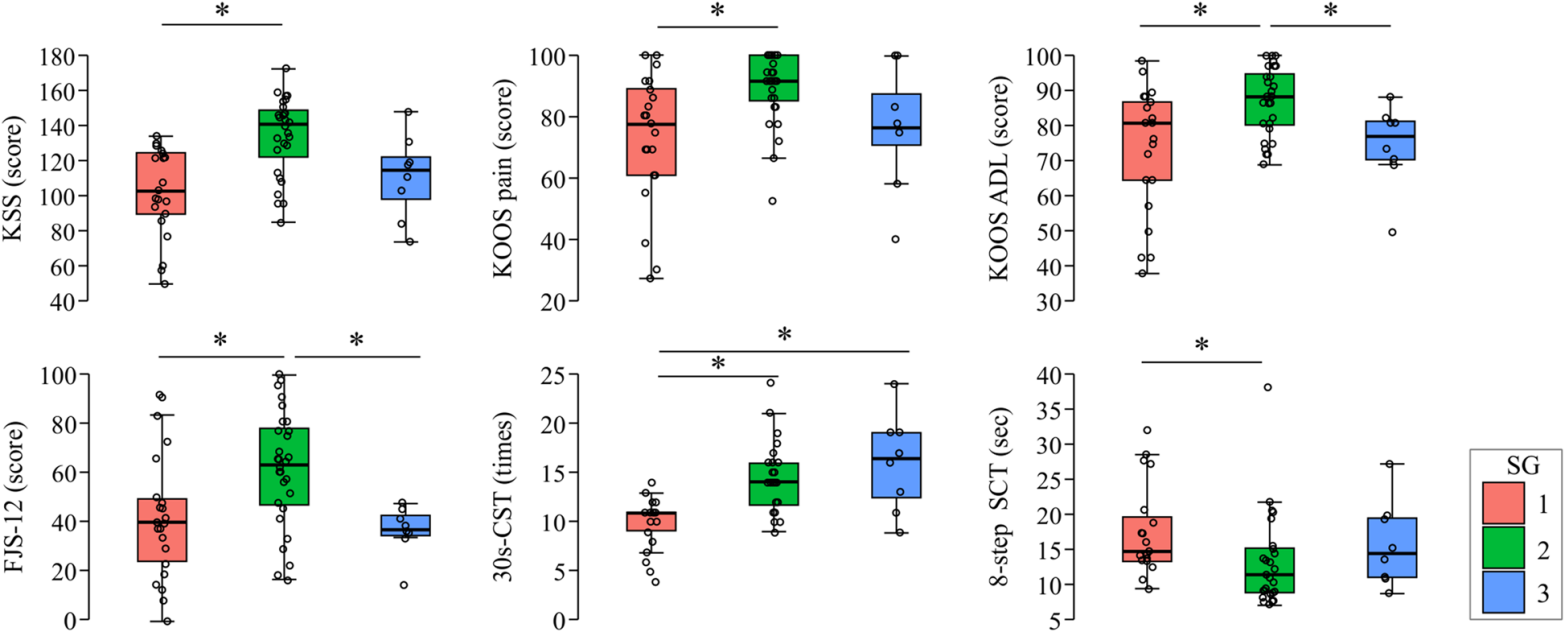
Box plots of items with significant differences. Outcomes showing significant differences between subgroups. SG1–SG3, first to third subgroup; KSS, Knee Society Score; KOOS, Knee Injury and Osteoarthritis Outcome Score; FJS-12, Forgotten Joint Score-12; 30 s-CST, 30-s Chair Stand Test; 8-step SCT, 8-step stair climbing test

The results of the one-way analysis of variance (ANOVA) are shown in Table 3, and the significantly different items are shown in Fig. 6. In the comparison between SG2 and SG3, SG2 showed significantly lower values for the FJS-12 and the KOOS ADL.

**Table 3:** Results of one-way analysis of variance

				**Statistical analysis (p)**		
	**SG1 (** ***n*** ** = 22)**	**SG2 (** ***n*** ** = 28)**	**SG3 (** ***n*** ** = 8)**	**SG1 × SG2**	**SG1 × SG3**	**SG1 × SG2**
PROMs						
KSS	103 [90–124]	141 [122.75–149]	114.5 [57.14–92.86]	< 0.05	NS	NS
KOOS						
Symptom	82.14 [57.14–92.86]	85.712 [78.57–96.43]	75 [64.29–81.25]	NS	NS	NS
Pain	77.78 [61.11–88.89]	91.67 [85.42–100]	76.39 [70.83–87.50]	< 0.05	NS	NS
Activity of daily life	80.88 [64.71–86.76]	88.24 [80.51–94.86]	77.21 [70.22–81.25]	< 0.05	NS	< 0.05
Sport/Recreation	25 [5–35]	42.5 [20–56.25]	22.5 [7.5–36.25]	NS	NS	NS
Quality of life	50 [37.5–75]	75 [62.5–81.25]	65.63 [62.5–81.25]	NS	NS	NS
FJS-12	39.79 [24.48–49.48]	63.54 [47.40–78.27]	37.5 [35.09–42.62]	< 0.05	NS	< 0.05
Performance test						
30 s-CST	11 [9.25–11]	14 [11.75–16]	16.5 [12.5–19]	< 0.05	< 0.05	NS
40mFPWT	33.88 [31.09–38.61]	30.50 [24.92–35.42]	31.49 [25.46–41.53]	NS	NS	NS
8-step SCT	14.72 [13.57–19.84]	11.6 [8.99–15.25]	14.52 [11.13–19.55]	< 0.05	NS	NS

Data are presented as median [IQR]. *SG* Subgroup, *PROMs* Patients reported outcome measures, *KSS* Knee Society Score, *KOOS* Knee Injury and Osteoarthritis Outcome Score, *FJS-12* Forgotten Joint Score-12; 30 s-CST, 30-s Chair Stand Test, *40mFPWT* 40 m fast-paced walk test, *8-step SCT* 8-step stair climbing test

## Discussion

Five principal components were extracted from the 48 kinematic parameters of STS, and their patterns were classified into three subgroups (SG) according to their kinematic characteristics. Patient classified as SG2, with kinematic characteristics like those of the momentum transfer (MT) strategy demonstrated in previous studies, was associated with better results in PROMs, and may be associated in particular with achieving a forgotten joint, which is considered the ultimate goal after TKA. This is the first study to quantitatively develop a kinematic evaluation of STS during the 30 s-CST through the use of markerless motion capture, an evaluation that has been performed subjectively by the therapist, and to clarify how results of the evaluation relate to clinical outcomes assessed in patients one year after TKA surgery.

### Principal component analysis

Two challenges need to be overcome to accomplish STS: first, one must shift the center of mass (COM) from a wide base of support (BOS) created by the hips, thighs, and feet to a narrow BOS of only the feet by anterior shank tilt after buttock release; and second, one must lift the COM from sitting height to standing height by generating forward momentum, primarily through trunk flexion, and upward momentum, primarily through lower-extremity extension movements [27]. PC1 was considered a kinematic parameter associated with the exertion of upward propulsion through anterior rotation of the thigh; achievement of STS requires afferent contraction of the hip and knee joint muscle groups that produce vertical propulsive force to lift the body [28]. A higher PC1 score indicates a greater contribution of hip and knee joint function to the upward shift of the center of gravity.

PC2 may reflect the angular velocity of trunk flexion, which generates forward energy in STS. The ability to exert sufficient muscle strength and coordination to generate upper-body motion before lifting the body is an important factor in achieving STS [28], and it has been reported that older adults and patients with knee OA have a greater trunk flexion angle and a tendency to project COM into the BOS during gluteal release [15, 29]. Higher PC2 scores were associated with a greater contribution of trunk flexion to the forward shift of the center of gravity.

PC3 is considered a kinematic parameter related to STS variability, representative of the consistency of repeated STS measurements. Regarding its significance, it could be that a lower PC3 score, indicating less variability in achieving STS, means better kinematic efficiency; however, less variability could also reflect a narrower ROM of the hip and knee joints. In a previous study, at one month post TKA, there was increased co-contraction of the quadriceps and hamstrings [12] and decreased ROM of the knee and hip joints [30] during STS. A higher PC3 score may indicate a greater range of joint motion during STS.

PC4 may indicate the ankle joint plantar-dorsiflexion angular velocity that contributes to the forward and upward energy generation in STS. As in the hip joint, the shank forward tilt motion affects the forward movement of the COM [31], and the forward energy is absorbed by the shank forward tilt movement and is converted to upward energy by the ankle joint plantar flexion movement [32]. The more the trunk is flexed, the more energy that is absorbed and converted into upward energy. The lower the trunk flexion movement, the more the shank forward tilt movement is required [33]. The faster the STS speed, the greater the increase in the ankle joint dorsiflexion moment [34]. A higher PC4 score is associated with a greater contribution of ankle joint function to the upward and forward movement of the center of gravity.

PC5 is considered a kinematic parameter associated with the action of switching from forward propulsion derived from hip and knee flexion motion into upward propulsion generated by trunk extension motion. In STS, both the scale and the timing of momentum generation are important [28], and both the magnitude of the antigravity movement needed to lift the COM upward and the timing of forward momentum generation are important in terms of energy efficiency. A higher PC5 score would indicate later timing of the momentum generation involved in the forward shift of the center of gravity through forward thigh rotation and greater contribution of trunk activity to upward momentum generation. The contribution of trunk activity was considered significant.

Various factors influence the determination of the STS motion strategy, but there is a trade-off between stability and force generation [17]. In healthy young adults, the MT strategy is selected as described by Hughes et al. [35]–[37]. The MT strategy is the most efficient method, although it requires instability, because it generates forward momentum through trunk flexion and raises the COM against gravity through coordinated extension of the lower extremities before the projected point of the COM enters the standing BOS. In contrast, the exaggerated trunk flexion (ETF) strategy, which is characterized by large trunk flexion in the elderly and knee OA patients, projects the COM into the BOS at the time of buttock release and does not utilize the rotational moment due to gravity in the early phase of the movement [27, 29]. Likewise, in the dominant vertical rise (DVR) strategy, which is characterized by trunk flexion that ceases immediately after hip release, the knee and hip joints are extended to allow vertical dominance of COM movement [35]. Both strategies have been reported to focus on stability rather than motor efficiency [29, 38, 39].

### Cluster analysis

For SG1, the fast angular velocities of trunk flexion and extension and slow angular velocities of hip and knee extension were the most characteristic. Older adults tend to flex their trunk more significantly before gluteal release, bringing the COM closer to the BOS and obtaining higher locomotion [40]. This strategy increases the hip flexion moment and decreases the knee flexion moment, thus decreasing the lower-extremity muscle strength required to lift the body upward [27]. The trunk flexion angle and knee joint extension muscle strength were inversely correlated during standing movements [41]. In addition, STS of patients one year after TKA surgery showed an increase in the angular velocity of knee extension compared to their preoperative level, but a decrease compared to that seen in healthy subjects [42] and an increase in hip flexion angle and extension moment on the operative side [10]. However, the postoperative STS exercise strategy of TKA patients is different from that of healthy participants, even one year after surgery. This exercise strategy has been interpreted as a compensation strategy to reduce the demand on knee extensor muscles, such as the quadriceps, but the characteristics of SG1, with high angular velocity of trunk movement and low angular velocity of lower limb movement, were thought to be like the ETF strategy shown in previous studies. The lower movement velocity was due to the emphasis on stability rather than efficiency, suggesting a lower functional reserve [40]. In particular, the present study regarded high speed as required for STS task execution, so use of this strategy strongly reflects a reduced functional reserve and constraints from the inability of this patient SG to select the optimal strategy for the task.

For SG2, characterized by fast maximum plantar and dorsiflexion angular velocities of the ankle joints and slow maximum angular velocity of trunk extension, the maximum angular velocity of hip and knee flexion was reached early in STS. In healthy subjects, the upper body moves before the body is lifted upward, and a motor strategy is chosen to convert the high forward momentum due to trunk flexion into upward work of raising the center of gravity toward the standing position [43]. In SG2, as in the able-bodied subject, forward rotation of the thighs by hip and knee joint motion early in STS generates sufficient forward momentum to accomplish the movement, and ankle joint motion controls the COM to convert forward momentum into vertical momentum without relying on trunk extension angular velocity. The fact that many of the items in SG2 had PC scores in the middle of their ranges compared to other SGs also suggests that this is a balanced strategy for trunk and limb locomotion in terms of speed and timing. This strategy is similar to the MT strategy shown in previous studies. In the MT strategy, the generation and conversion of momentum in the upper body and the body as a whole reduces the load on lower-extremity muscle strength and trunk movement, but body balance becomes unstable during the transition period when momentum is converted. This is because the COM is often located behind the trailing edge of the foot-only BOS immediately after the foot is released, and the section of the BOS transition after release is the most challenging for COM control owing to the backward rotational moment that occurs [27]. This is why STS at the maximum speed presents the most difficulty for COM control. Based on the above, the MT strategy, which emphasizes force generation rather than stability, is considered the optimal locomotor strategy for this research task, which requires STS tasks at maximum speed, and SG2, which utilizes the MT strategy, may indicate a high level of functional reserve capacity.

For SG3, the characteristics are generally in contrast to those of SG1, with less trunk and ankle motion, and higher hip and knee extension angular velocities. In STS, the trunk acts to generate and control momentum, but as the body ages, the maximum trunk angle becomes smaller. In addition, patients with knee OA are unable to fully transfer the forward momentum generated by trunk flexion to the lower extremities, resulting in inefficient movement strategies that utilize energy flow [38]. To compensate for the above, a movement strategy that relies on hip and knee joint muscle output was selected [29] that was like the DVR strategy described in previous studies when used at the maximum speed required for the 30 s-CST. It is seen as a more desirable movement strategy than the ETF, but it should be noted that PC1 is very high and PC2 is very low compared to in other SGs. In other words, the COM is lifted upward with the trunk relatively vertical, and the forward momentum cannot be converted to upward momentum, resulting in significantly larger knee joint maximal torque values during STS than in the other movement strategies [29]. Therefore, it is a strategy that places a greater load on the lower limb for the generation of upward momentum and that is inferior to the MT strategy in terms of kinetic efficiency of force generation.

### Difference in clinical outcome

Regardless of advances in surgical techniques, one in five patients feel unsatisfied after TKA [44, 45], and the factors necessary to achieve good satisfaction after TKA are unclear. Surgical techniques (implant design [46, 47], postoperative alignment [48], and surgical approach [49]) had no influence and were significantly correlated with postoperative knee function [50], and postoperative motion function may influence clinical outcomes.

In this study, more items were significantly lower in SG1 than in SG2 with respect to PROMs; however, SG1 and SG3 did not show significant differences. Patients in SG1 chose the ETF strategy for STS in the 30 s-CST compared to SG2, who chose the MT strategy. We hypothesized that patients in SG1, showed decreased functional reserve capacity, which may have manifested itself as difficulties experienced by the patient in activities of daily living. In addition, SG2 and SG3 did not show significant differences in the 30 s-CST scores, but SG2 scored significantly higher on the FJS-12 and KOOS ADL. The difference between SG2 and SG3 may be due to the fact that SG2 chose a strategy that placed a greater burden on the hip and knee joints.

The most important point of this study is that SG2 was significantly higher than the other SGs in FJS-12, which is an index for achieving the ultimate goal of the “forgotten joint”, which is living without joint awareness, in post-TKA patients. The MT strategy is the preferred STS strategy because the ETF and DVR strategies require extra motion and joint torque [29]. SG2, who were able to choose the MT strategy for STS in the 30 s-CST, may have had a better ability to choose the appropriate exercise strategy for the environment and tasks in activities of daily living other than STS, resulting in better subjective patient assessment performance. Thus, we hypothesized that STS kinematics of the 30 s-CST may capture physical functions that cannot be captured by the 30 s-CST score alone. Our results suggest that kinematic differences in STS may be expressed as differences in FJS-12, which is an important finding for rethinking physical therapy to improve knee function and satisfaction after TKA. We believe that the results of this study are useful because the responsibility of physical therapy is not only to enable or disable movement but also to increase freedom to adapt to any environment, prevent secondary disability, and return to society with reacquired efficient posture and movement strategies.

An important aspect of this study is that the kinematic parameters were not analyzed individually, rather a cluster analysis was performed based on values obtained by extracting the principal components from the kinematic parameters in STS and reducing their dimensions. As a result, the kinematic parameters necessary to achieve STS were integrated, such as "PC1: Kinematic parameters related to upward propulsive force exerted by thigh forward rotation," and then classified by the size of the components, rather than by the target angles or angular velocities of individual joint motions. Motion classification was performed. In clinical practice, the acquisition of joint angles does not necessarily lead to improved movement; this requires an analysis of how the body is able to move in the actual task to determine treatment guidelines. As an example, SG1 was characterized by low PC1. In order to improve it, it was necessary to practice STS in a sitting posture in a raised position using an elevating bed, and to practice lowering the sitting surface step by step in an environment that tends to cause femoral anteversion, and to practice STS with a therapist stabilizing the distal femur with one hand on the knee joint and the other hand on the hip joint and guiding the patient in the direction of anterior rotation from the proximal femur. While treatment strategy decisions in clinical practice have often been based on the experience of individual therapists, the use of markerless motion capture to clarify the underlying kinematic mechanisms of functional impairment may contribute to the formulation of new treatment strategies in physical therapy for patients with TKA. The use of markerless motion capture has the potential to contribute to the formulation of new treatment strategies in physical therapy for patients with TKA.

### Limitations

This study had some limitations. It should be noted that STS during the 30 s-CST was cut out and evaluated in this study and does not reflect the STS performed in daily life activities. It has been reported that STS during the 5-chair stand test and STS performed in daily life have different motor strategies [51]. However, it has not been clarified how the exercise objective, an important factor in the selection of movement strategy, is optimized in daily life. The exercise objective is determined based on the weighting of various factors such as energy cost, safety, and pain avoidance, and the exercise strategy is selected accordingly [18]. The following is a list of some of the most important factors in exercise selection. Since the goal required for the 30 s-CST is to achieve STS at maximal velocity, we believe that the results of this study are useful to assess whether the patient has the ability to choose a velocity-weighted exercise strategy. Therefore, it is important to interpret the results of this study as an evaluation of physical function in TKA patients rather than focusing solely on STS improvement. Furthermore, it is important to consider the fact that the study is based on sagittal plane movement strategies alone; therefore, it is only a limited representation of the STS movements employed by one-year postoperative TKA patients when performing the 30 s-CST. In a systematic review of kinematic changes in standing movements [16], it was stated that in order to fully capture the changes in STS after TKA, it is necessary to analyze the forehead and horizontal plane, rather than limiting the analysis to the kinematics and kinetics of the knee in the sagittal plane. This is an item that we believe should have been considered in this study. It has been reported that patients with knee osteoarthritis have trunk lateral flexion to the less-affected side [15], and in the 30 s-CST, where high performance at maximal velocity is required, it would be desirable to add this motion to the evaluation items, considering the possibility that the preoperative exercise strategy may remain after the surgery. We believe that it is desirable to add an endpoint to consider the possibility that preoperative STS strategies may remain after surgery. The limitation of this study is that only one direction of analysis could be performed because video capture was performed with a single camera, with an emphasis on clinical simplicity. However, there are reports of 3D analysis using markerless motion capture [52] that we would like to clarify in future studies.

Another issue is the low cumulative contribution ratio of 55.7% resulting from the principal component analysis, which means that the extracted principal components may not provide a good overview of the kinematic data. Although a higher cumulative contribution ratio is desirable because it more strongly reflects the kinematic data information used in the analysis, the criteria for the contribution ratio are not clearly defined. One way to address this is to select many principal components to increase the cumulative contribution ratio. However, we considered that this not only makes it difficult to capture features when a cluster analysis is performed but also runs the risk of biasing the classification due to overlearning. Furthermore, reducing the data on which principal component analysis was performed might also be considered. However, since no previous study has kinematically analyzed the STS of the 30 s-CST by markerless motion capture, we considered it difficult to clearly determine which kinematic data were unnecessary.

We also believe that more detailed and accurate classification may be possible by using dynamic, muscular variables in the analysis. Previous studies on STS have shown that coactivation between the quadriceps and hamstrings occurs in patients with knee osteoarthritis [30] and that muscle activation of the quadriceps and floor half strength of the operated limb are decreased after TKA [12]. The use of dynamic, muscular variables in this study may have provided a more detailed and accurate analysis. However, the use of electromyography for analysis requires the application of electrodes to the skin, which is inconsistent with the concept of this study, which aims to reduce the burden on patients for clinical application, and was therefore excluded. Although it should be noted that only approximately half of the kinematic parameters used were reflected in this study, we believe that the content extracted supports many previous studies using 3D motion analysis devices. This is an important finding that demonstrates the usefulness of markerless motion capture, which can be easily used in clinical practice. Finally, this study was only a kinematic assessment and did not identify the interventions that were effective. Although the results of this study showed that SG2, who selected a movement strategy similar to the MT strategy, performed better on clinical outcomes, this does not mean that it is preferable to aim for acquisition of the movement strategy of SG2 as normal movement, since effective movement strategies vary depending on the environment and task. Because movement is repetitive, future research should clarify that, when considering treatment content, the underlying physical function of individuals who are able to select MT strategies should be considered in evaluation of the STS of the 30 s-CST.

## Conclusions

Patient use of movement strategy SG2, with kinematic characteristics like those of the MT strategy demonstrated in previous studies, was associated with better results in PROMs, and may be associated with achieving a forgotten joint, which is considered the ultimate goal after TKA. This study is the first to demonstrate that kinematic differences in STS can translate into differences in clinical outcomes, which is an important finding for rethinking physical therapy to improve knee function and satisfaction after TKA.

## Supplementary Information

**Additional file 1.**

## Data Availability

The datasets used and/or analyzed during the current study are available from the corresponding author on reasonable request.

## Abbreviations

8-step SCT: 8-Step stair climbing test
30 s-CST: 30-Second Chair Stand Test
40 m FPWT: 40 M fast-paced walk test
ANOVA: One-way analysis of variance
BMI: Body mass index
BOS: Base of support
COM: Center of mass
DVR: Dominant vertical rise
ETF: Exaggerated trunk flexion
FJS-12: Forgotten Joint Score-12
FTA: Femorotibial angle
KOOS: Knee Injury and Osteoarthritis Outcome Score
KSS: Knee Society Score
MT: Momentum transfer
OARSI: Osteoarthritis Research Society International
PC1-PC5: The first-fifth principal component
PROMs: Patients reported outcome measures
ROM: Range of Motion
SG: Subgroup
STS: Sit-to-stand
TKA: Total knee arthroplasty
