# Supplementary material for: Clinical utility of markerless motion capture for kinematic evaluation of sit-to-stand during 30 s-CST at one year post total knee arthroplasty: a retrospective study
Source: BMC Musculoskelet Disord. 2023 Apr 1;24:254. doi: 10.1186/s12891-023-06364-3 (PMC10067213; doi:10.1186/s12891-023-06364-3)
Supplement: Supplementary file 1 — Additional file 1. [file 12891_2023_6364_MOESM1_ESM.docx]

**Supplemental Data 1.** Reliability and reproducibility of calculation of joint angles during 30s-CST using Pose-cap

|  | Inter-rater reliability | | Intra-rater reliability | |
| --- | --- | --- | --- | --- |
| Target Joint | ICC (1,1) | 95% CI | ICC (2,1) | 95% CI |
| Trunk | 0.49 | ［0.48-0.50］ | 1.00 | ［1.00-1.00］ |
| Hip | 0.74 | ［0.72-0.75］ | 1.00 | ［1.00-1.00］ |
| Knee | 0.80 | ［0.0-0.81］ | 1.00 | ［1.00-1.00］ |
| Ankle | 0.11 | ［-0.10-0.30］ | 0.99 | ［0.99-0.99］ |

Six healthy subjects (age :21 ± 0.82 y) were enrolled. Reliability was calculated by comparison with a three-dimensional motion analyzer (VICON). Reproducibility was calculated using the video data taken. ICC, Intraclass correlation coefficients.
